# Treatments, prognostic factors, and genetic heterogeneity in advanced cholangiocarcinoma: A multicenter real‐world study

**DOI:** 10.1002/cam4.6892

**Published:** 2024-03-08

**Authors:** Alessandro Ottaiano, Mariachiara Santorsola, Anna Diana, Andrea Belli, Maria Luisa Lentini Graziano, Jessica Orefice, Renato Patrone, Annabella Di Mauro, Giosuè Scognamiglio, Fabiana Tatangelo, Mario De Bellis, Mauro Piccirillo, Francesco Fiore, Salvatore Stilo, Luca Tarotto, Marco Correra, Sara Di Lorenzo, Maurizio Capuozzo, Antonio Avallone, Lucrezia Silvestro, Antonella Bianco, Vincenza Granata, Piera Federico, Vincenzo Montesarchio, Bruno Daniele, Francesco Izzo, Guglielmo Nasti

**Affiliations:** ^1^ Istituto Nazionale Tumori di Napoli, IRCCS “G. Pascale” Napoli Italy; ^2^ Medical Oncology Unit Ospedale del Mare Napoli Italy; ^3^ Medical Oncology Unit AORN Ospedali dei Colli‐Monaldi‐Cotugno‐CTO Napoli Italy; ^4^ Coordinamento Farmaceutico ASL‐Naples‐3 Ercolano Italy

**Keywords:** cholangiocarcinoma, genetics, prognostic factors, real‐word study, tumor heterogeneity

## Abstract

**Background and Aims:**

Cholangiocarcinoma (CCA), a rare and aggressive hepatobiliary malignancy, presents significant clinical management challenges. Despite rising incidence and evolving treatment options, prognosis remains poor, motivating the exploration of real‐world data for enhanced understanding and patient care.

**Methods:**

This multicenter study analyzed data from 120 metastatic CCA patients at three institutions from 2016 to 2023. Kaplan–Meier curves assessed overall survival (OS), while univariate and multivariate analyses evaluated links between clinical variables (age, gender, tumor site, metastatic burden, ECOG performance status, response to first‐line chemotherapy) and OS. Genetic profiling was conducted selectively.

**Results:**

Enrolled patients had a median age of 68.5 years, with intrahepatic tumors predominant in 79 cases (65.8%). Among 85 patients treated with first‐line chemotherapy, cisplatin and gemcitabine (41.1%) was the most common regimen. Notably, one‐third received no systemic treatment. After a median 14‐month follow‐up, 81 CCA‐related deaths occurred, with a median survival of 13.1 months. Two clinical variables independently predicted survival: response to first‐line chemotherapy (disease control vs. no disease control; HR: 0.27; 95% CI: 0.14–0.50; *p* < 0.0001) and metastatic involvement (>1 site vs. 1 site; HR: 1.99; 95% CI: 1.04–3.80; *p* = 0.0366). The three most common genetic alterations involved the *ARID1A*, *tp53*, and *CDKN2A* genes.

**Conclusions:**

Advanced CCA displays aggressive clinical behavior, emphasizing the need for treatments beyond chemotherapy. Genetic diversity supports potential personalized therapies. Collaborative research and deeper CCA biology understanding are crucial to enhance patient outcomes in this challenging malignancy.

## 1 INTRODUCTION

Cholangiocarcinoma (CCA), in its extrahepatic (involving the biliary tree within the hepatoduodenal ligament) and intrahepatic forms (within the liver parenchyma), represents 2–3% of gastrointestinal tumors and 10%–15% of all hepatobiliary malignancies.^1^ It derives from cholangiocytes of the intra or extrahepatic tract. The majority of cases are sporadic forms. Recognized risk factors are primary and secondary sclerosing cholangitis, typhus, certain infections (Opisthochis viverrini and Clonorchis sinensis), exposure to X‐ray contrast media, and chronic biliary inflammatory‐associated conditions. Surgical resection, where possible, is the only curative treatment. In fact, the clinical signs of a CCA occur mainly when it is already in an advanced stage (the most frequently involved organ is the liver followed by lymphnodes, lungs, and bone).^2^ Biliary stenting and/or biliary drainage via bypass are the most common early active symptom control (ASC) therapeutic interventions, especially in patients presenting with jaundice.^1^, ^2^

The interest in this cancer is increasing because of a slight but significant and constant increase in incidence, and the low response to chemotherapy. In fact, over the past three decades, the incidence rates of intrahepatic forms have increased in Western Europe and Japan compared to Eastern countries.^3^ The subtle symptomatology often leads to a delay in diagnosis and only one in five patients can undergo radical surgical resection. Most patients present with locally advanced or metastatic disease, primarily involving local lymph nodes, peritoneum, and liver.^1^, ^2^, ^3^

Chemotherapy is the most commonly used therapeutic option in the metastatic phase. Based on the results of the phase III randomized ABC‐02 study, the combination of cisplatin and gemcitabine has become the standard first‐line treatment, demonstrating a median overall survival of 11.7 months compared to 8.1 months in patients treated with gemcitabine alone (HR: 0.64; 95% CI: 0.52–0.80; *p* < 0.001).^4^ Very recently, the TOPAZ‐1 trial demonstrated a significant reduction in the risk of death with the use of anti‐PD‐L1 durvalumab in combination with cisplatin and gemcitabine compared to placebo plus chemotherapy (HR: 0.80; 95% Confidence Interval: 0.66–0.97; *p* = 0.021).^5^ These findings have resulted in the approval of cisplatin plus gemcitabine and durvalumab by the FDA and European Medicines Agency (EMA) as the new standard of care in the first‐line treatment for previously untreated unresectable or metastatic CCA. In the second‐line setting, a phase 3 study (ABC‐06) was recently published, demonstrating a survival advantage with FOLFOX (folinic acid, oxaliplatin, and fluorouracil) as a second‐line therapy compared to ASC in patients who progressed after receiving the standard first‐line treatment with cisplatin and gemcitabine. The median overall survival was 6.2 months in the ASC plus FOLFOX group compared to 5.3 months in the ASC‐only group (HR: 0.69; 95% CI: 0.50–0.97; *p* = 0.031).^6^ However, the treatment of metastatic CCA in clinical practice also relies on the use of tailored forms of chemotherapy that have been selected in numerous studies, largely undersized and retrospective (irinotecan, taxanes, fluoropyrimidines, etc.), showing variable response rates ranging from 0% to 22%.^7^

As in other types of cancer, recent studies have revealed genetic alterations underlying the transformation and progression of CCA. The most frequently observed mutations in CCA include *ARID1A*, *TP53*, *KRAS*, *CDKN2A*/*B*, *FGFRs*, *IDH1*, and *SMAD4*.^8^ Based on this knowledge, it is possible to inhibit the altered function of these genes with targeted molecular therapies. Indeed, recently, infigratinib^9^ and pemigatinib^10^ have been FDA‐approved for use in pretreated tumors with FGFR2 fusions or mutations. Furthermore, for patients with *IDH1* mutations refractory to one or two lines of systemic therapy, the *IDH1* inhibitor ivosidenib showed efficacy and received FDA approval in this clinical setting.^11^ Other potential therapeutic targets include *BRAF* (p.V600E) alterations, *NTRK* fusions, and *HER2* amplifications.^12^

Since the neoplasm, although on the rise, is rare, it is valuable to collect data from multiple centers, especially those with a high level of expertise, in order to conduct a comprehensive real‐world practice study that can provide deeper insights into the management and outcomes of this condition, ultimately enhancing our understanding and improving patient care.

## 2 METHODS

### 2.1 Study design and data source

This is a retrospective, multicenter, real‐world study conducted using data extracted from a database that contains clinical information on metastatic CCA patients treated at three institutions in Naples, Italy (from 2016 to 2023): (1) Istituto Nazionale dei Tumori di Napoli, IRCCS “G. Pascale” (Structure of Innovative Therapies for Abdominal Metastases), (2) Ospedale del Mare, and (3) Azienda Osepdaliera dei Colli. Patients in this study were treated based on the decisions of multidisciplinary tumor boards (MTB), reached through consensus discussions and following the ESMO guidelines.^13^ Inclusion in this cohort was not subject to specific selection criteria. The primary outcome of this study was to describe overall survival (OS) with respect to various clinical variables. The study was conducted in adherence to the principles outlined in the Declaration of Helsinki, and all patients provided signed informed consent before undergoing any of the treatments or participating in the genetic tests described in this article.

### 2.2 Patients management

Within the framework of the MTBs, treatment choices were thoroughly reviewed, encompassing input from medical oncologists, surgical oncologists, radiation oncologists, pathologists, radiologists, and genetic counselors. Patients underwent regular follow‐up, including total body computed tomography (CT) scans conducted every 3 months, or more frequently if clinically indicated due to disease progression. Treatment response was assessed following the RECIST (Response Evaluation Criteria In Solid Tumors) guidelines.^14^ Specifically, a complete response (CR) indicated the complete disappearance of all detectable signs of the disease on total body CT scans. A partial response (PR) was characterized by a minimum of a 30% reduction in the combined diameters of target lesions. Stable disease (SD) encompassed cases with tumor size changes falling between a 30% decrease and a 20% increase. Progressive disease (PD) was diagnosed when there was a minimum 20% increase in the combined diameters of target lesions.

### 2.3 Statistical analyses and data presentation

This study primarily maintains a descriptive nature, with its main focus on assessing and describing differences in overall survival (OS) based on selected variables, including age, gender, primary tumor localization, metastatic involvement, PS ECOG, and response to first‐line chemotherapy. The primary outcome measures OS, calculated from the time of advanced disease diagnosis until death from any cause. Given the heterogeneity in progression assessment (variation in radiologic monitoring after clinical progression became evident or conducted in diverse institutions), progression‐free survival was not a study objective. In this context, vital status stands as the most robust and dependable outcome for analysis.

The Kaplan–Meier product limit method was employed to illustrate OS curves. Univariate analysis's statistical significance was determined using a two‐tailed Log‐Rank test. Multivariate analysis was employed to explore prognostic interactions between OS and covariates, utilizing the Cox proportional‐hazards regression model. Survival probability estimates for different dichotomized covariates were expressed as Hazard Ratios (HRs), representing the risk of an event (death) at any given time for a patient with the risk factor present compared to a patient with the risk factor absent, assuming all other covariates are identical. A significance level of *p* < 0.05 denoted statistical significance. HRs were accompanied by 95% confidence intervals (CIs) in all analyses. Statistical analyses were conducted using Excel software and MedCalc® version 20.112 (MedCalc Software Ltd, Ostend, Belgium).

## 3 RESULTS

Table 1 reports the demographic and clinical features of the analyzed patients. The median age was 68.5 years, with an age range between 39 and 89 years. Regarding gender distribution, 68 patients (56.6%) were female, and 52 patients (43.3%) were male. The primary tumor site was predominantly intrahepatic in 79 cases (65.8%), followed by extrahepatic in 41 cases (34.2%, with 30 located perihilar and 11 distal). Supplementary File 1 contains a list of metastatic sites in all patients. Performance status according to ECOG (Eastern Cooperative Oncology Group) was distributed as follows: 80 patients (66.6%) had a PS ECOG score of 0 or 1, 11 patients (9.2%) had a score of 2, and 29 patients (24.2%) had a score of 3. Jaundice was noted as the onset symptom in 27 patients (22.5%), while 82 patients (68.3%) presented without jaundice, and 11 patients (9.2%) had unknown onset symptoms. Surgery for the primary tumor was performed in 45 patients (37.5%) as part of their treatment, with 7 of them (15.5%) undergoing surgery after receiving chemotherapy, and 38 patients (84.4%) undergoing surgery before commencing chemotherapy. The vast majority of patients (95.8%) did not receive prior adjuvant therapy. A total of 73 patients (60.8%) presented with more than two metastatic sites. Among the 85 patients treated with first‐line chemotherapy, the most commonly administered treatments were cisplatin and gemcitabine (41.1%), and oxaliplatin and gemcitabine (30.6%). In the realm of second‐line therapies, fluorouracil and irinotecan or oxaliplatin, as well as gemcitabine and capecitabine, were the most frequently employed options (27.5%, 20.0%, and 20.0%, respectively). In terms of the number of treatment lines, 45 patients (37.5%) received only a single line of treatment, 24 patients (20.0%) received two lines of treatment, 16 patients (13.3%) received more than two lines of treatment, and 35 patients (29.2%) did not receive any treatment. The response to first‐line chemotherapy varied, with over half of the patients experiencing progressive disease, underscoring the inherently aggressive nature of CCA from a biological perspective.

**TABLE 1:** Clinico‐pathological characteristics of patients.

Variable	No.	%
Age		
Median, range (year)	68.5 (39–89)	
Gender		
Female	68	56.6
Male	52	43.3
Primary tumor site		
Intrahepatic	79	65.8
Extrahepatic	41	34.2
PS ECOG		
0/1	80	66.6
2	11	9.2
3	29	24.2
Onset with jaundice		
No	82	68.3
Yes	27	22.5
Unknown	11	9.2
Surgery		
No	75	62.5
Yes	45	37.5
After chemotherapy	7	15.5
Before chemotherapy	38	84.4
Previous adjuvant therapy		
No	115	95.8
Yes	5	4.16
No. of metastatic sites		
1	47	39.2
≥2	73	60.8
Type of first‐line treatment		
Cisplatin and gemcitabine	35	41.1
Oxaliplatin and gemcitabine	26	30.6
Gemcitabine	11	12.9
Cisplatin, gemcitabine, and Durvalumab	6	7.1
Gemcitabine and capecitabine	3	3.5
Fluoropirimidines and oxaliplatin	2	2.3
Cisplatin, gemcitabine, atezolizumab, and bevacizumab	1	1.2
Capecitabine	1	1.2
Type of second‐line treatment		
Fluorouracile and irinotecan	11	27.5
Fluoropirimidines and oxaliplatin	8	20.0
Gemcitabine and capecitabine	8	20.0
Capecitabine	4	10.0
Pemigatinib	3	7.5
Atezolizumab	1	2.5
Cisplatin and gemcitabine	1	2.5
Gemcitabine	1	2.5
Fluorouracile	1	2.5
Cisplatin and fluorouracile	1	2.5
Gemcitabine and fluorouracile	1	2.5
No. of treatment lines		
1	45	37.5
2	24	20.0
>2	16	13.3
None	35	29.2
Best response to first‐line CT^a^		
Progressive disease	45	54.2
Stable disease	20	24.1
Partial response	16	19.3
Complete response	2	2.4

^a^ The response was not assessable in two patients.

**FIGURE 1:**
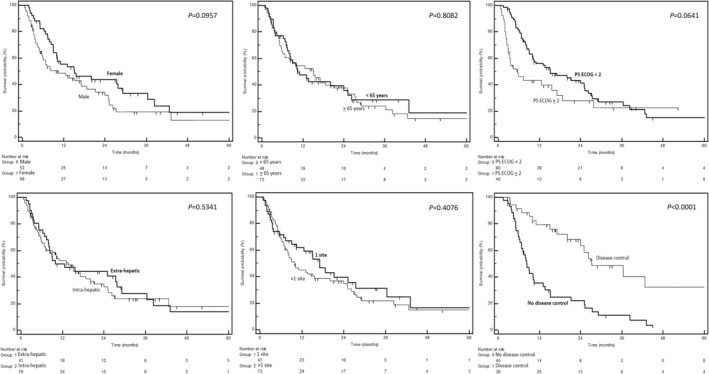
Kaplan–Meier curves depicting overall survival, stratified by potential prognostic factors (as indicated in each figure). The *p*‐values from the log‐rank test are presented alongside each curve.

After a median follow‐up period of 14 months, there were 81 deaths related to CCA. We conducted a comprehensive analysis to evaluate potential prognostic factors that could impact overall survival in our cohort. These variables were selected through a consensus discussion among the authors (the selected variables were: age, gender, localization of the primary tumor, metastatic involvement, PS ECOG, and response to first‐line chemotherapy). The temporal survival trends based on dichotomized variables are depicted in the Kaplan–Meier curves shown in Figure 1. The results of both uni‐ and multivariate analyses are summarized in Table 2. In the multivariate analysis, only two clinical variables exhibited significant associations with survival: the response to first‐line chemotherapy (disease control vs no disease control; HR: 0.27; 95% CI: 0.14–0.50; *p* < 0.0001) and metastatic involvement (>1 site vs 1 site; HR: 1.99; 95% CI: 1.04–3.80; *p* = 0.0366). The onset with jaundice and the presence of type II diabetes (T2D) were selected as two clinical variables with potential prognostic significance; however, patients with jaundice at onset or T2D did not exhibit a significantly different survival trend compared to those who presented with normal bilirubin levels or were not affected by T2D (Supplementary File 2). Since 45 patients were genetically characterized as part of tests conducted to assess their eligibility for clinical trials or based on individual case discussions at the MTBs, we conducted a descriptive analysis of pathogenetically significant genetic events. The three most frequent alterations involved the *ARID1A*, *tp53*, and *CDKN2A* genes (Supplementary File 3).

**TABLE 2:** Uni‐ and multivariate analysis for overall survival.

Co‐variate	Dicothomization	Median survivals (months)	No. of events/patients	*p* at univariate	HR	95% CI	*p* at multivariate	Adjusted HR	95% CI
**Age**	≥65 years vs <65 years	15.1 vs 10.4	49/72 vs 32/48	0.8082	0.94	0.60–1.48	0.8446	1.05	0.60–1.85
Gender	M vs F	10.4 vs 15.1	47/68 vs 34/52	0.0957	1.45	0.93–2.26	0.2250	1.43	0.80–2.55
Localization	Intra vs Extrahepatic	13.6 vs 12.6	52/79 vs 29/41	0.5341	1.15	0.73–1.81	0.4151	1.28	0.70–2.32
Metastatic involvement	>1 site vs 1 site	9.9 vs 17.1	53/73 vs 28/47	0.4076	1.20	0.77–1.89	0.0366	1.99	1.04–3.80
PS ECOG	<2 vs ≥2	15.1 vs 5.8	55/80 vs 26/40	0.0614	0.60	0.36–1.02	0.2413	2.50	0.53–11.68
Response to first‐line CT	DC vs no DC	27.2 vs 8.3	18/38 vs 39/45	<0.0001	0.30	0.17–0.53	<0.0001	0.27	0.14–0.50

Abbreviations: CI, confidence interval; DC, disease control; F, female; HR, hazard ratio.

The incidence of the most common genetic alterations, compared to what is described in the scientific literature,^15^ is reported in Table 3.

**TABLE 3:** Incidence of the most common genetic mutations.

Genes	Observed incidence %	Reported incidence %^a^
*ARID1A*	20.0	25.0
*Tp53*	20.0	35.0
*CDKN2A*	17.7	15.0
*CDKN2B*	13.3	15.0
*KRAS*	13.3	20.0
*FGFR2*	11.1	25.0
*BAP1*	11.1	15.0

*Note*: The mutations with an incidence in more than 3 patients (out of the 45 characterized) are presented. The complete mutational landscape is detailed in Supplementary File 3. However, given the therapeutic significance of *IDH1* mutations, it is noteworthy that they were present in 3 patients, accounting for an incidence of 6.6%, as opposed to the 15.0% reported in the literature (^a^see Capuozzo et al.^15^). Incidence is calculated as the number of positive cases for a pathogenic mutation divided by the total number of characterized patients.

## 4 DISCUSSION

CCA poses significant challenges in the field of oncology, especially in its advanced stages. Our study is based on real‐world data, combining experiences from three high‐volume institutions. It has descriptively analyzed the clinical and prognostic determinants characterizing the disease in its advanced stage in 120 patients, as well as the genetic landscape in a subset of them. Through rigorous analysis and collaborative efforts, two important messages have emerged, highlighting the complexities associated with managing advanced CCA.

First and foremost, advanced CCA displays an aggressive clinical behavior. Remarkably, in clinical practice, approximately one‐third of the patients were unable to undergo any systemic medical treatment (35 out of 120 total patients). Our median survival data (median: 13.1 months) compare favorably with what has been previously reported in the scientific literature from past retrospective studies (medians: 10–14 months), both in unselected populations for specific treatments^16^, ^17^, ^18^ and in patients who received only cisplatin and gemcitabine therapies.^19^ This data likely suggests the challenge of achieving a substantial survival improvement with chemotherapy alone. Furthermore, our comprehensive multivariate analysis has identified two primary prognostic factors that significantly influence patient outcomes: the response to first‐line chemotherapy and the extent of tumor burden, measured by the number of involved metastatic sites. These findings reaffirm the aggressive and elusive nature of CCA. Significantly, 29% of patients in our case series did not undergo any specific form of treatment. The primary rationale behind this decision stemmed from the compromised clinical condition of the patients, coupled with the rapid and aggressive trajectory of the disease. The choice to forego treatment in these cases was primarily influenced by the acknowledgment that the patients were in poor health, making them unsuitable candidates for therapeutic interventions. In such instances, the clinical assessment indicated that the potential benefits of treatment might be outweighed by the risks and the potential deterioration of the patients' overall well‐being. However, even in the presence of treatment heterogeneity, the response to first‐line chemotherapy remains a pivotal determinant of survival, accentuating the immediate demand for effective treatment strategies. Furthermore, the importance of tumor burden, even when quantified approximately by the number of metastatic sites, should not be underestimated. This metric serves not only as a valuable prognostic marker but also underscores the necessity for comprehensive disease assessment. It emphasizes the need to devise therapeutic interventions targeting multifocal disease progression.

Investigations regarding the efficacy of cisplatin and gemcitabine compared to alternative regimens have not been attempted, both because the scientific inquiry would be futile (cisplatin and gemcitabine currently constitute the standard chemotherapy backbone) and because, in the present case series derived from real‐world practice, the population treated with “alternative” chemotherapy regimens exhibited a significantly older age and poorer overall clinical conditions compared to those treated with cisplatin and gemcitabine. In addition, given the retrospective non‐randomized nature of the study, efficacy comparisons would be unjustifiable also from a descriptive standpoint as well.

An interesting revelation from our study is the pronounced genetic heterogeneity of the CCA. No genetic event exceeded an incidence of 20% in our cohort (the most frequent altered genes were: *ARID1A* 20.0% of cases, *TP53* 20.0%, and *CDKN2A* 17.7%). This observation underscores the exceptional heterogeneity of CCA, which presents distinct challenges for diagnosis and treatment. This heterogeneity suggests that a one‐size‐fits‐all approach to therapy may not suffice for these patients. Instead, CCA represents a paradigm where individualized treatment plans, guided by genetic profiling, may offer a more promising avenue to enhance patient outcomes. Understanding the genetic foundations of CCA is pivotal for the development of targeted therapies addressing the specific vulnerabilities of each patient.

In 2022, the most extensive international observational study on the management of CCA was published, encompassing 2234 patients from 11 European countries as documented in the ENSCCA registry.^20^ This study features a thorough analysis, providing distinct prognostic insights into intrahepatic, perihilar, and distal forms. Within the patient cohort, 49.6% had unresectable disease, necessitating the application of active palliative interventions, including locoregional therapy, chemotherapy, or a combination thereof. The median overall survival in this subgroup was 10.6 months, contrasting with the 4.0 months observed in individuals receiving best supportive care (20.6%). In our specific case series, a median overall survival of 15.1 months was observed in patients undergoing chemotherapy, while those receiving best supportive care exhibited 5.6 months. These differences may be inherently linked to the variability in sample size, the diverse geographical origins of the patients, and the heterogeneity in the administration of initial therapeutic interventions.

Our study also confirms the escalating incidence of CCA, particularly the intrahepatic form. Although a definitive explanation remains elusive, factors such as environmental exposures and evolving risk profiles necessitate further investigation.^21^, ^22^ Despite significant progress in recent years and the availability of a standard first‐line treatment, managing advanced CCA remains a complex challenge, compounded by the low response rates to standardized therapeutic regimens. Given the relatively infrequent occurrence of CCA cases, the collection of multicenter data is imperative. Collaborative efforts among institutions are crucial for pooling resources and data to enhance our understanding of the disease, inform study design, and refine treatment strategies.

Two additional clinical factors were explored: the initial onset of the disease with jaundice, which in some studies has been highlighted as a positive prognostic factor,^23^ and the presence of type 2 diabetes (T2D), which, in other solid neoplasms, appears to be a negative prognostic factor^24^, ^25^ (Supplementary File 2). In both cases, we did not find statistically significant differences. This observation is intriguing, although it may be contingent upon the limitations of our study, which we are about to describe for a proper interpretation of the data.

In fact, our study does have certain limitations that need to be acknowledged. First and foremost, the retrospective nature of the study imposes inherent limitations. In a retrospective study, data are collected from past records, which may be incomplete or subject to biases. This could potentially lead to missing or incomplete data, as well as the inability to control variables as effectively as in prospective studies. Additionally, retrospective studies are observational and results may be influenced by unmeasured confounding factors. Despite these limitations, retrospective studies provide valuable insights into real‐world clinical practice and outcomes. Another notable limitation of our study is the relatively small cohort size, particularly among those who underwent genetic characterization. However, when considering the incidence of CCA, these limitations are somewhat intrinsic to the field. CCA is a rare cancer, and assembling prospective large cohorts for in‐depth studies can be challenging. Therefore, while our study sample size may be limited, it is representative of the challenges researchers face when studying this rare disease.

In addition to the above limitations, other considerations should be taken into account. Variability in treatment regimens and practices across different institutions and regions may introduce some degree of heterogeneity in our cohort. Furthermore, the evolving landscape of CCA management, including the introduction of targeted therapies and immunotherapies, may not be fully reflected in our study due to its retrospective and real‐word nature.

Despite these limitations and considerations, our study contributes valuable insights into the management of advanced CCA, shedding light on the disease aggressiveness, genetic heterogeneity, and the need for personalized treatment approaches. Further research, collaboration, and prospective studies are warranted to continue advancing our understanding and management of this challenging malignancy. In fact, the aggressive and enigmatic biology of CCA calls for innovative research and clinical approaches. Even if a few cases of our series underwent to targeted‐therapies, identifying novel therapeutic targets and developing precision medicine interventions hold promise for improving patient outcomes in CCA. Sustained investigations into the molecular and genetic underpinnings of CCA are essential to drive advancements in diagnosis and therapy.

Several pragmatic considerations can guide clinicians in their approach to genetic assessments. In instances where the patient is young (below 50 years of age) or demonstrates good overall health (PS ECOG 0 or 1), it is advisable to conduct a comprehensive gene panel analysis before commencing treatment. Ideally, this panel should encompass multigene NGS assessments, including *IDH1*, *FGFR2*, *HER2*, and *NTRK* rearrangements.^13^, ^26^ Despite the lower frequency of the latter in CCA, its association with elevated response rates justifies consideration. For other mutations (*ARID1A*, *KRAS*, *PIK3CA*, *tp53*, etc.), in CCA, the therapeutic implications remain unclear. However, recognizing these mutations may prove beneficial for facilitating prompt enrollment in potential clinical trials. In situations where patients present compromised clinical conditions but are still deemed appropriate for chemotherapy, a comprehensive evaluation of their genetic profile becomes indispensable, especially considering the observed clinical improvement following the timely initiation of chemotherapy. It is noteworthy that the plastic and evolving nature of CCA emphasizes the adaptability in genetic assessments. It is evident that this approach raises economic and ethical considerations beyond the scope of this study. However, in recognizing the dynamic landscape of CCA, we advocate for a balanced perspective on the timing of genetic assessments. While an initial evaluation may be expedient, the inherently changing nature of the disease leads us to recommend, at the very least, a repeat assessment in close proximity to the initiation of targeted therapies, especially in cases where the interval between genetic evaluation and the commencement of biologic therapy exceeds 1 year. In this context, liquid biopsies clearly offer a non‐invasive and easily accessible alternative for performing genetic assessments, particularly given the methodological challenges associated with obtaining solid biopsies in cases of CCA.

Beyond these considerations, we stress the significance of a personalized approach to patient care. This involves integrating genetic assessments into the broader clinical context (age, PS ECOG, comorbidities, patient expectations, etc.), considering the patient's overall health, and monitoring treatment responses. Collaborative efforts with genetic counselors and molecular pathologists are crucial to ensure appropriate genetic testing and accurate interpretation of genetic results.

In conclusion, our real‐world study contributes to describe the intricacies of managing this aggressive disease. The significance of the response to first‐line chemotherapy and tumor burden cannot be overstated, underscoring the imperative for innovative treatment strategies. Furthermore, the genetic diversity within CCA highlights the critical role of a personalized medicine approach in guiding future therapeutic decisions. As we navigate the challenges posed by this heterogeneous neoplasm, collaborative research endeavors and a deeper comprehension of its biology are pivotal in shaping the future landscape of CCA management.

## AUTHOR CONTRIBUTIONS

**Alessandro Ottaiano:** Conceptualization (equal); formal analysis (equal); investigation (equal); methodology (equal); resources (equal); software (equal); writing – original draft (equal). **Mariachiara Santorsola:** Conceptualization (equal); data curation (equal); formal analysis (equal); methodology (equal); software (equal); writing – original draft (equal). **Anna Diana:** Investigation (equal); validation (equal); writing – review and editing (equal). **Andrea Belli:** Investigation (equal). **Maria Luisa Lentini Graziano:** Investigation (equal). **Jessica Orefice:** Investigation (equal). **Renato Patrone:** Investigation (equal). **Annabella Di Mauro:** Investigation (equal). **Giosuè Scognamiglio:** Investigation (equal). **Fabiana Tatangelo:** Investigation (equal). **Mario de Bellis:** Investigation (equal). **Mauro Piccirillo:** Investigation (equal). **Francesco Fiore:** Investigation (equal). **Salvatore Stilo:** Investigation (equal). **Luca Tarotto:** Investigation (equal). **Marco Correra:** Investigation (equal). **Sara Di Lorenzo:** Investigation (equal). **Maurizio Capuozzo:** Investigation (equal). **Antonio Avallone:** Investigation (equal). **Lucrezia Silvestro:** Investigation (equal). **Antonella Bianco:** Investigation (equal). **Vincenza Granata:** Investigation (equal). **Piera Federico:** Investigation (equal). **Vincenzo Montesarchio:** Investigation (equal). **Bruno Daniele:** Investigation (equal). **Francesco Izzo:** Conceptualization (equal); formal analysis (equal); investigation (equal); resources (equal); supervision (equal); writing – original draft (equal). **Guglielmo Nasti:** Conceptualization (equal); data curation (equal); investigation (equal); methodology (equal); supervision (equal); writing – original draft (equal).

## FUNDING INFORMATION

This work was partially supported by grants from the Italian Government, Ministry of Health (www.salute.gov.it, accessed on October 11, 2023), Ricerca Corrente 2022 L4/8.

## CONFLICT OF INTEREST STATEMENT

The authors declare that they have no conflict of interest.

## INSTITUTIONAL REVIEW BOARD STATEMENT

Ethical review and approval by the Comitato Etico Campania 1 were not required for retrospective studies on human participants in accordance with institutional requirements.

## INFORMED CONSENT STATEMENT

Patients provided signed informed consent prior to undergoing any treatments and genetic assessments.

## Supporting information


Supplementary File 1.

Supplementary File 2.

Supplementary File 3.

## Data Availability

The data used in this study are available upon reasonable request from the corresponding author. Examples of source clinical and genetic data are reported at https://zenodo.org/records/10362105.

## References

[1] Khan SA , Tavolari S , Brandi G . Cholangiocarcinoma: epidemiology and risk factors. Liver Int. 2019;39(Suppl 1):19‐31. doi:10.1111/liv.14095 30851228

[2] Blechacz B . Cholangiocarcinoma: current knowledge and new developments. Gut Liver. 2017;11:13‐26. doi:10.5009/gnl15568 27928095 PMC5221857

[3] Valle JW , Kelley RK , Nervi B , Oh DY , Zhu AX . Biliary tract cancer. Lancet. 2021;397:428‐444. doi:10.1016/S0140-6736(21)00153-7 33516341

[4] Valle J , Wasan H , Palmer DH , et al. ABC‐02 trial investigators. Cisplatin plus gemcitabine versus gemcitabine for biliary tract cancer. N Engl J Med. 2010;362:1273‐1281. doi:10.1056/NEJMoa0908721 20375404

[5] Oh DY , He AR , Qin S , et al. For the TOPAZ‐1 investigators. Durvalumab plus gemcitabine and cisplatin in advanced biliary tract cancer. NEJM Evid. 2022;1:1. doi:10.1056/EVIDoa2200015 38319896

[6] Lamarca A , Palmer DH , Wasan HS , et al. Second‐line FOLFOX chemotherapy versus active symptom control for advanced biliary tract cancer (ABC‐06): a phase 3, open‐label, randomised, controlled trial. Lancet Oncol. 2021;22:690‐701. doi:10.1016/S1470-2045(21)00027-9 33798493 PMC8082275

[7] Ying J , Chen J . Combination versus mono‐therapy as salvage treatment for advanced biliary tract cancer: a comprehensive meta‐analysis of published data. Crit Rev Oncol Hematol. 2019;139:134‐142. doi:10.1016/j.critrevonc.2019.01.001 30979533

[8] Lamarca A , Hubner RA , David Ryder W , Valle JW . Second‐line chemotherapy in advanced biliary cancer: a systematic review. Ann Oncol. 2014;25:2328‐2338. doi:10.1093/annonc/mdu162 24769639

[9] Javle M , Lowery M , Shroff RT , et al. Phase II study of BGJ398 in patients with FGFR‐altered advanced cholangiocarcinoma. J Clin Oncol. 2018;36:276‐282. doi:10.1200/JCO.2017.75.5009 29182496 PMC6075847

[10] Abou‐Alfa GK , Sahai V , Hollebecque A , et al. Pemigatinib for previously treated, locally advanced or metastatic cholangiocarcinoma: a multicentre, open‐label, phase 2 study. Lancet Oncol. 2020;21:671‐684. doi:10.1016/S1470-2045(20)30109-1 32203698 PMC8461541

[11] Abou‐Alfa GK , Macarulla T , Javle MM , et al. Ivosidenib in IDH1‐mutant, chemotherapy‐refractory cholangiocarcinoma (ClarIDHy): a multicentre, randomised, double‐blind, placebo‐controlled, phase 3 study. Lancet Oncol. 2020;21:796‐807. doi:10.1016/S1470-2045(20)30157-1 32416072 PMC7523268

[12] Manne A , Woods E , Tsung A , Mittra A . Biliary tract cancers: treatment updates and future directions in the era of precision medicine and Immuno‐oncology. Front Oncol. 2021;11:768009. doi:10.3389/fonc.2021.768009 34868996 PMC8634105

[13] Vogel A , Bridgewater J , Edeline J , et al. Biliary tract cancer: ESMO clinical practice guideline for diagnosis, treatment and follow‐up. Ann Oncol. 2023;34:127‐140. doi:10.1016/j.annonc.2022.10.506 36372281

[14] Eisenhauer E , Therasse P , Bogaerts J , et al. New response evaluation criteria in solid tumours: revised RECIST guideline (version 1.1). Eur J Cancer. 2009;45:228‐247. doi:10.1016/j.ejca.2008.10.026 19097774

[15] Capuozzo M , Santorsola M , Landi L , et al. Evolution of treatment in advanced cholangiocarcinoma: old and new towards precision oncology. Int J Mol Sci. 2022;23:15124. doi:10.3390/ijms232315124 36499450 PMC9740631

[16] Seung SJ , Saherawala H , Syed I , Shephard C , Clouthier DL , Chen E . Real‐world treatment patterns and survival outcomes for treated biliary tract cancer patients using administrative databases in Ontario. J Gastrointest Oncol. 2023;14:1806‐1816. doi:10.21037/jgo-23-155 37720427 PMC10502530

[17] Maeda O , Ebata T , Shimokata T , et al. Chemotherapy for biliary tract cancer: real‐world experience in a single institute. Nagoya J Med Sci. 2020;82:725‐733. doi:10.18999/nagjms.82.4.725 33311803 PMC7719462

[18] Koshiol J , Yu B , Kabadi SM , Baria K , Shroff RT . Epidemiologic patterns of biliary tract cancer in the United States: 2001‐2015. BMC Cancer. 2022;22:1178. doi:10.1186/s12885-022-10286-z 36384474 PMC9670575

[19] Kim BJ , Hyung J , Yoo C , et al. Prognostic factors in patients with advanced biliary tract cancer treated with first‐line gemcitabine plus cisplatin: retrospective analysis of 740 patients. Cancer Chemother Pharmacol. 2017;80:209‐215. doi:10.1007/s00280-017-3353-2 28597043

[20] Izquierdo‐Sanchez L , Lamarca A , La Casta A , et al. Cholangiocarcinoma landscape in Europe: diagnostic, prognostic and therapeutic insights from the ENSCCA registry. J Hepatol. 2022;76:1109‐1121. doi:10.1016/j.jhep.2021.12.010 35167909

[21] Sirica AE , Gores GJ , Groopman JD , et al. Intrahepatic cholangiocarcinoma: continuing challenges and translational advances. Hepatology. 2019;69:1803‐1815. doi:10.1002/hep.30289 30251463 PMC6433548

[22] Javle M , Lee S , Azad NS , et al. Temporal changes in cholangiocarcinoma incidence and mortality in the United States from 2001 to 2017. Oncologist. 2022;27:874‐883. doi:10.1093/oncolo/oyac150 35972334 PMC9526482

[23] Lleo A , Colapietro F , Maisonneuve P , et al. Risk stratification of cholangiocarcinoma patients presenting with jaundice: a retrospective analysis from a tertiary referral center. Cancers (Basel). 2021;13:2070. doi:10.3390/cancers13092070 33922972 PMC8123266

[24] Ottaiano A , Circelli L , Santorsola M , et al. Metastatic colorectal cancer and type 2 diabetes: prognostic and genetic interactions. Mol Oncol. 2022;16:319‐332. doi:10.1002/1878-0261.13122 34668636 PMC8763648

[25] Christou N , Bergen ES , Canton C , et al. Impact of diabetes and metformin use on recurrence and outcome in stage II‐III colon cancer patients‐a pooled analysis of three adjuvant trials. Eur J Cancer. 2022;166:100‐111. doi:10.1016/j.ejca.2022.02.005 35279470

[26] Mosele F , Remon J , Mateo J , et al. Recommendations for the use of next‐generation sequencing (NGS) for patients with metastatic cancers: a report from the ESMO precision medicine working group. Ann Oncol. 2020 Nov;31(11):1491‐1505. doi:10.1016/j.annonc.2020.07.014 32853681

