# Supplementary material for: Treatments, prognostic factors, and genetic heterogeneity in advanced cholangiocarcinoma: A multicenter real‐world study
Source: Cancer Med. 2024 Mar 8;13(4):e6892. doi: 10.1002/cam4.6892 (PMC10923031; doi:10.1002/cam4.6892)
Supplement: Supplementary file 1 [file CAM4-13-e6892-s001.pdf]

Supplementary File 1. List of metastatic sites in all patients.

Patient_ID	Metastatic_sites
1	Peritoneum
2	Liver
3	Liver, lungs
4	Liver
5	Liver
6	Liver
7	Lungs, peritoneum, bone
8	Liver
9	Liver
10	Liver, peritoneum
11	Liver
12	Liver, lymphnodes
13	Lungs, bone, lymphnodes
14	Liver
15	Liver, lymphnodes
16	Liver, bone
17	Liver, peritoneum
18	Liver, peritoneum
19	Liver, lungs, peritoneum
20	Liver, peritoneum
21	Liver
22	Liver, peritoneum
23	Liver, lungs, bone, lymphnodes
24	Liver
25	Liver, adrenal gland
26	Liver, peritoneum
27	Liver, lungs, peritoneum

Patient_ID	Metastatic_sites
28	Liver
29	Liver, lymphnodes
30	Liver, peritoneum, bone
31	Liver, lungs, bone
32	Liver
33	Liver
34	Liver
35	Liver, lungs, bone
36	Liver, lungs, lymphnodes
37	Liver, lungs, brain
38	Liver, lungs, lymphnodes
39	Liver, lymphnodes
40	Liver, peritoneum
41	Liver
42	Lungs, bone, lymphnodes
43	Liver
44	Liver, lungs
45	Liver, bone
46	Liver, lungs, lymphnodes
47	Liver, peritoneum, lymphnodes
48	Liver
49	Liver, peritoneum, lymphnodes, bone, uterus
50	Liver, lymphnodes
51	Liver
52	Liver, lymphnodes
53	Liver, lymphnodes
54	Liver, lymphnodes

Patient_ID	Metastatic_sites
55	Liver
56	Liver, peritoneum
57	Liver, lymphnodes
58	Liver, lungs, lymphnodes
59	Liver
60	Liver, lungs, peritoneum
61	Liver, peritoneum
62	Lymphnodes
63	Liver, peritoneum, lymphnodes
64	Liver, lungs, lymphnodes
65	Liver, lungs, peritoneum
66	Liver, peritoneum, lymphnodes
67	Liver, peritoneum, lymphnodes
68	Liver
69	Lymphnodes
70	Liver, lymphnodes
71	Liver, lungs
72	Liver
73	Liver, peritoneum
74	Liver, peritoneum
75	Liver, peritoneum
76	Liver
77	Liver, bone, peritoneum
78	Liver, lungs
79	Liver, lungs, lymphnodes
80	Liver, peritoneum
81	Liver

Patient_ID	Metastatic_sites
82	Liver, peritoneum
83	Liver
84	Liver
85	Lymphnodes
86	Peritoneum
87	Liver
88	Pancreas
89	Liver, pancreas, kidney
90	Liver, peritoneum
91	Liver, lungs, lymphnodes
92	Liver, lymphnodes
93	Liver, lungs
94	Liver, lungs, lymphnodes, spleen
95	Liver, lymphnodes
96	Liver
97	Peritoneum
98	Peritoneum
99	Lungs, peritoneum, lymphnodes
100	Liver, lungs, peritoneum
101	Liver, lungs
102	Liver, lungs
103	Liver, lungs
104	Liver
105	Liver
106	Liver
107	Liver
108	Liver

Patient_ID	Metastatic_sites
109	Liver, lungs
110	Liver
111	Liver
112	Liver, lungs, lymphnodes, peritoneum
113	Liver
114	Liver, lungs, lymphnodes
115	Peritoneum
116	Lungs, lymphnodes
117	Peritoneum
118	Liver
119	Liver, lymphnodes
120	Liver, lungs
