# Supplementary material for: Treatments, prognostic factors, and genetic heterogeneity in advanced cholangiocarcinoma: A multicenter real‐world study
Source: Cancer Med. 2024 Mar 8;13(4):e6892. doi: 10.1002/cam4.6892 (PMC10923031; doi:10.1002/cam4.6892)
Supplement: Supplementary file 2 [file CAM4-13-e6892-s002.pdf]

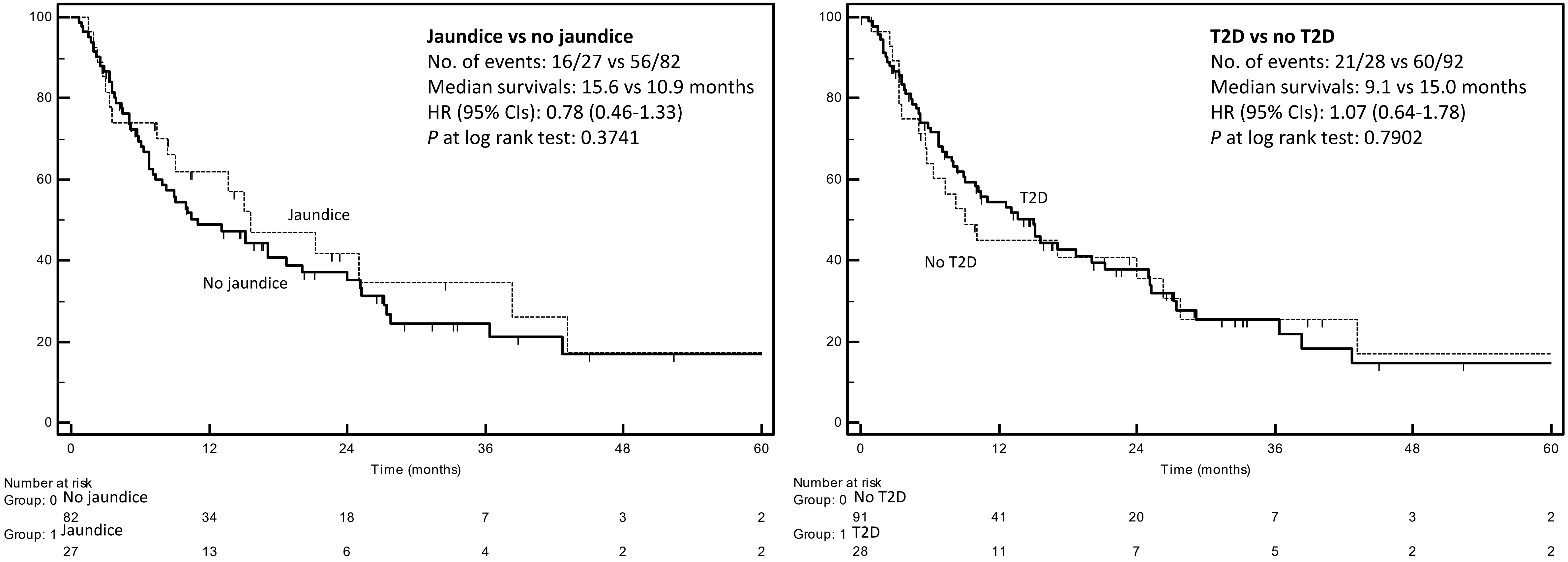

Supplementary File 1. The Kaplan-Meier survival curves for the clinical cohort are depicted based on the presence or absence of jaundice at the onset of CCA and the presence or absence of type 2 diabetes (T2D). The figures include median survival times, Hazard Ratios (HR) with associated Confidence Intervals (CIs), and p-values derived from the Log Rank test. Information regarding the onset of jaundice was unavailable for 11 patients, and the presence or absence of diabetes was not reported for 1 patient.
