# Supplementary material for: Treatments, prognostic factors, and genetic heterogeneity in advanced cholangiocarcinoma: A multicenter real‐world study
Source: Cancer Med. 2024 Mar 8;13(4):e6892. doi: 10.1002/cam4.6892 (PMC10923031; doi:10.1002/cam4.6892)
Supplement: Supplementary file 3 [file CAM4-13-e6892-s003.pdf]

**Supplementary File 2.** Genetic landscape of genes altered in the analyzed clinical series.

The majority of genetic tests were performed using the Next Generation Sequencing Technique (NGS), specifically employing the FoundationOne CDx® panel (accessed via https://www.accessdata.fda.gov/cdrh_docs/pdf17/P170019S006C.pdf, last accessed on September 15, 2023). Only a minor subset (6 patients) underwent testing with alternative gene panels (TSO500®, Oncomine Solid Tumour DNA kit®), which were complemented by targeted methodologies to investigate additional disease-specific genetic alterations, such as IDH1/2 mutations and FGFR2 fusions. Some of these tests also served as screening for potential enrollment in clinical studies. The data from these patients are clearly anonymized, and we are not authorized to disclose the specific mutations found to avoid violating strict requirements on data security, sharing, and patient consent. Some of these restrictions are also related to intellectual property and commercial interests. In essence, specific findings, such as rare mutations or pathogenic mutations, have the potential to jeopardize intellectual or commercial interests when exposed to competitors or the public. This occurs in this retrospective study due to the lack of a predetermined plan for genetic testing and its unequivocal and focused utilization toward a specific objective. As a result, a judicious balance has been achieved between scientific information and data dissemination by exclusively presenting three categories of alterations (mutation, amplification, loss), as agreed upon with the companies involved. The information provided, although summarized, illustrative, and aggregated, remains clinically relevant and actionable.
In summary, while the sharing of genetic data from oncology patients is valuable for advancing research and personalized medicine, various factors, including privacy concerns, legal obligations, and commercial interests, particularly in retrospective and multicentric studies, can limit the extent to which such data is disseminated in specific, detailed, and non-aggregated forms. Balancing these considerations is essential to ensure that the potential benefits of data sharing are realized while safeguarding patient rights and data integrity.

In a green background, the genes that have been altered in more than three patients are highlighted.

| **Acronym** | **Full name** | **No. of patients** |
|---|---|---|
| ACVR1B | Activin A Receptor Type 1B | |
| | Mutation | 1 |
| ALK | Anaplastic Lymphoma Kinase | |
| | Mutation | 2 |
| ARID1A | AT-rich interaction domain 1A | |
| | Mutation | 9 |
| AKT2 | AKT Serine/Threonine Kinase 2 | |
| | Amplification | 1 |
| ASXL1 | Additional Sex Combs Like 1 | |
| | Mutation | 2 |
| AXL | Anexelecto | |
| | Amplification | 1 |
| | Mutation | 1 |
| ATM | Ataxia Telangiectasia Mutated | |
| | Mutation | 1 |
| BAP1 | BRCA1-associated protein 1 | |
| | Mutation | 5 |
| BCL2L1 | BCL2-Like 1 | |
| | Amplification | 1 |
| BCOR | BCL6 Corepressor | |
| | Mutation | 1 |
| BCORL1 | BCL6 Corepressor Like 1 | |
| | Mutation | 1 |

| | | |
|---|---|---|
| BRAF | v-raf murine sarcoma viral oncogene homolog B1 | |
| | Mutation | 2 |
| BRCA1 | BReast CAncer gene 1 | |
| | Mutation | 2 |
| BRIP1 | BRCA1-Interacting Protein 1 | |
| | Mutation | 1 |
| CCNE1 | Cyclin E1 | |
| | Amplification | 1 |
| CCND1 | Cyclin D1 | |
| | Amplification | 2 |
| CDKN2A | Cyclin-dependent kinase inhibitor 2A | |
| | Loss | 6 |
| | Mutation | 2 |
| CDKN2B | Cyclin-dependent kinase inhibitor 2B | |
| | Loss | 6 |
| CDK12 | Cyclin-dependent kinase 12 | |
| | Amplification | 1 |
| CDK6 | Cyclin-dependent kinase 6 | |
| | Amplification | 1 |
| CEBPA | CCAAT/enhancer-binding protein alpha | |
| | Amplification | 1 |
| CSF3R | Colony-stimulating factor 3 receptor | |
| | Mutation | 1 |
| CTNNB1 | Catenin Beta 1 | |
| | Mutation | 1 |
| EMSY | BRCA2-interacting transcriptional repressor | |
| | Mutation | 1 |
| EP300 | E1A-binding protein p300 | |
| | Mutation | 1 |
| EPHA3 | Ephrin type-A receptor 3 | |
| | Amplification | 1 |
| EPHB4 | Ephrin receptor B4 | |
| | Mutation | 1 |
| | Amplification | 1 |
| ERBB3 | Erb-B2 receptor tyrosine kinase 3 | |
| | Mutation | 1 |
| EZH2 | Enhancer of zeste homolog 2 | |
| | Mutation | 1 |
| FGFR2 | Fibroblast growth factor receptor 2 | |
| | Mutation | 1 |
| | Fusion | 4 |
| | FGFR2-INA | 2 |
| | FGFR2- KIAA1598 | 1 |
| | FGFR2-BICC1 | 1 |
| FGF3 | Fibroblast growth factor 3 | |
| | Amplification | 2 |
| FGF4 | Fibroblast growth factor 4 | |
| | Amplification | 2 |
| FGF10 | Fibroblast growth factor 10 | |
| | Amplification | 1 |
| FGF19 | Fibroblast growth factor 19 | |
| | Amplification | 2 |
| FLCN | Folliculin | |
| | Mutation | 1 |
| GNAS | Guanine Nucleotide-Binding Protein G(s) Alpha Subunit | |
| | Mutation | 1 |

| | | |
|---|---|---|
| GPC3 | Glypican 3 | |
| | Mutation | 1 |
| HER2 | Human epidermal growth factor receptor 2 | |
| | Amplification | 2 |
| | Mutation | 1 |
| HGF | Hepatocyte Growth Factor | |
| | Amplification | 1 |
| IDH1/2 | Isocitrate dehydrogenase 1/2 | |
| | Mutation | 3 |
| IFNGR1 | Interferon Gamma Receptor 1 | |
| | Mutation | 1 |
| IRF4 | Interferon Regulatory Factor 4 | |
| | Mutation | 1 |
| JAK1 | Janus Kinase 1 | |
| | Mutation | 1 |
| JAK2 | Janus Kinase 2 | |
| | Mutation | 1 |
| KDM6A | Lysine Demethylase 6A | |
| | Loss | 1 |
| KDR | Kinase Insert Domain Receptor | |
| | Mutation | 1 |
| KRAS | Kirsten rat sarcoma viral oncogene homolog | |
| | Mutation | 6 |
| LRP1B | Low-Density Lipoprotein Receptor-Related Protein 1B | |
| | Mutation | 1 |
| LYN | Lck/Yes-Related Novel Protein Tyrosine Kinase | |
| | Amplification | 1 |
| MAP2K4 | Mitogen-Activated Protein Kinase Kinase 4 | |
| | Loss | 1 |
| MDM2 | Mouse Double Minute 2 Homolog | |
| | Amplification | 2 |
| MERTK | MER Proto-Oncogene, Tyrosine Kinase | |
| | Mutation | 1 |
| MITF | Microphthalmia-Associated Transcription Factor | |
| | Mutation | 1 |
| MLH1 | MutL Homolog 1 | |
| | Loss | 1 |
| MLL | Mixed Lineage Leukemia | |
| | Mutation | 2 |
| MLL2 | Mixed Lineage Leukemia 2 | |
| | Mutation | 1 |
| MST1R | Macrophage Stimulating 1 Receptor | |
| | Mutation | 1 |
| MTAP | Methylthioadenosine Phosphorylase | |
| | Loss | 3 |
| MTOR | Mechanistic Target of Rapamycin (serine/threonine kinase) | |
| | Mutation | 1 |
| MUTYH | MutY DNA Glycosylase | |
| | Mutation | 1 |
| MYC | Myelocytomatosis oncogene | |
| | Amplification | 1 |
| NF1 | Neurofibromin 1 | |
| | Mutation | 3 |
| NF2 | Neurofibromin 2 | |
| | Loss | 1 |
| NOTCH1 | Neurogenic Locus Notch Homolog Protein 1 | |

| | | |
|---|---|---|
| | Mutation | 1 |
| NOTCH3 | Neurogenic Locus Notch Homolog Protein 3 | |
| | Mutation | 1 |
| NRAS | Neuroblastoma RAS Viral Oncogene Homolog | |
| | Mutation | 1 |
| | Amplification | 1 |
| PARP1 | Poly(ADP-ribose) Polymerase 1 | |
| | Mutation | 1 |
| PBRM1 | Polybromo 1 | |
| | Mutation | 2 |
| PIK3CA | Phosphatidylinotitol 3-kinase catalytic subunit alpha | |
| | Mutation | 1 |
| PDCD1LG2 | Programmed Cell Death 1 Ligand 2 | |
| | Mutation | 1 |
| PDGFRA | Platelet-Derived Growth Factor Receptor alpha | |
| | Mutation | 1 |
| PDGFRB | Platelet-Derived Growth Factor Receptor Beta | |
| | Mutation | 1 |
| PDK1 | 3-Phosphoinositide-Dependent Protein Kinase 1 | |
| | Mutation | 1 |
| PMS2 | PMS1 Homolog 2 | |
| | Mutation | 1 |
| PTEN | Phosphatase and Tensin Homolog | |
| | Mutation | 1 |
| PPP2R1A | Protein phosphatase 2regulatorysubunitA | |
| | Mutation | 1 |
| RAD21 | RAD21 Cohesin Complex Component | |
| | Mutation | 1 |
| | Amplification | 1 |
| RB1 | Retinoblastoma Protein 1 | |
| | Mutation | 3 |
| RBM10 | RNA Binding Motif Protein 10 | |
| | Mutation | 1 |
| RET | Rearranged During Transfection | |
| | Fusion | |
| | RET-GPHN fusion | 1 |
| RICTOR | Rapamycin-Insensitive Companion of mTOR | |
| | Mutation | 1 |
| | Amplification | 1 |
| RNF43 | Ring Finger Protein 43 | |
| | Mutation | 2 |
| SF3B1 | Splicing Factor 3B Subunit 1 | |
| | Mutation | 1 |
| SMAD4 | Mothers against decapentaplegic homolog 4 | |
| | Mutation | 2 |
| | Loss | 1 |
| SMARCA4 | SWI/SNF-Related Matrix-Associated Actin-Dependent Regulator of Chromatin Subfamily A Member 4 | |
| | Mutation | 1 |
| SPEN | Spen Family Transcriptional Repressor | |
| | Mutation | 1 |
| TERT | Telomerase Reverse Transcriptase | |
| | Mutation | 1 |
| TET2 | Tet Methylcytosine Dioxygenase 2 | |
| | Mutation | 1 |
| TGFBR2 | Transforming Growth Factor Beta Receptor 2 | |

| | | |
|---|---|---|
| | Mutation | 1 |
| TP53 | Tumor protein 53 | |
| | Mutation | 8 |
| | Loss | 1 |
| ZNF703 | Zinc Finger Protein 703 | |
| | Amplification | 1 |
| WHSC1L1 | Wolf-Hirschhorn Syndrome Candidate 1-Like 1 | |
| | Amplification | 1 |
